# Short-Term Relocation Stress-Induced Hematological and Immunological Changes in *Saimiri boliviensis boliviensis*

**DOI:** 10.1155/2021/5318590

**Published:** 2021-11-08

**Authors:** Pramod N. Nehete, Bharti P. Nehete, Akash G. Patel, Sriram Chitta, Henrieta Scholtzova, Lawrence E. Williams

**Affiliations:** ^1^Department of Comparative Medicine, The University of Texas MD Anderson Cancer Center, Bastrop, TX, USA; ^2^The University of Texas Graduate School of Biomedical Sciences, Houston, TX, USA; ^3^Center for Cognitive Neurology and Department of Neurology, New York University Grossman School of Medicine, New York, NY, USA

## Abstract

Nonhuman primates are frequently transported to a new location or temporarily relocated within their colony. Both transportation and relocation expose animals to new environments, causing them to undergo a stress response (before adapting). In our NHP colony, the mentioned situations are not infrequent for many reasons, including maintenance. The objective of this study was to determine whether abrupt changes consisting of relocation, housing, separation, and grouping could influence hematological and immunological parameters and thereby functional activity. The current study used squirrel monkeys as a model to investigate the stress-inducing effects of relocation within a facility, while animals acclimated to new situations (physical, housing). A detailed blood analysis revealed significant changes in lymphocytes, triglycerides, total protein, creatinine, and ALT. Flow cytometric analysis of peripheral blood showed reduction in CD3^+^, CD4^+^, and CD8^+^ T cells and monocytes, while B cells and natural killer (NK) cells changed with relocation. Simultaneously, changes in functional activity of immune cells altered proliferative responses and as shown by ELISpot (IFN *γ*). Though the parameters studied are not affected as severely as those in animals transported by road or air, stress responses induced by intrafacility relocation are significant and worth consideration. Our findings indicate that squirrel monkeys mimic the features seen in humans exposed to social stressors and may serve an important model for understanding the mechanisms of stress-induced immune dysfunction in humans.

## 1. Introduction

Previously, we have shown immunological and hematological changes due to long distance relocation of colonies of chimpanzees [1], cynomolgus macaques [2], rhesus macaques [3], and squirrel monkeys [4]. Stress in adult NHPs can be caused by psychosocial events in which animals are subjected to disruptive interactions within their established social circuits. One known way to induce stress in NHPs is to relocate the animal from one facility to another [5–7]. In or within captive housing colonies, NHPs will form social connections to housing mates. However, because of experimental, breeding, and other requirements, captive NHPs are often shifted from one social group to another. It is known that relocation of animals from one social housing cohort into individual caging or the introduction of unfamiliar conspecifics is especially stressful. The resulting stress can be detected as increased levels of plasma and urinary cortisol, loss of appetite, and an initial period of subdued physical activity [8–11].

Many human studies have shown that social stressors are associated with changes in the immune system, which decrease a patient's ability to fight diseases including cancer and caused by infections. Studies investigating the relationship between stress and survivorship in breast cancer [12, 13] found that increased stress (anger, repression, and hopelessness) leads to a decreased survival of patients [14, 15].

Furthermore, cancer therapies are more successful when patients are comfortable in their environment [16–18]; hence, there appears to be a direct relationship between survivability and levels of stress [13]. Relocation stress syndrome, familiar in human social settings, is characterized by depressed mood, anxiety, social withdrawal, and somatic symptoms that may be accompanied by transient HPA activation [19–22]. Loss of a family member has been associated with an increased risk for somatic and psychiatric somatic illness and dysregulation of the HPA axis as well [23–26]. Social support helps to dampen the negative effects of stressors such as relocation or separation [27, 28].

The squirrel monkey model has been used as an experimental model for several human diseases such as in malaria vaccine development, opportunistic human experimental transmission of Creutzfeldt-Jakob disease (CJD), other transmissible spongiform encephalopathies, endemic viruses, and Alzheimer's disease [29, 30]. In order to improve our understanding of the effects of social group relocation on the immune system, we used squirrel monkeys as a model system and assessed various immune and functional parameters that may be influenced by short-term relocation within the facility while initiating a new research study.

## 2. Materials and Methods

### 2.1. Animals

#### 2.1.1. Monkey Care and Housing

The studies were performed in elderly (14-15 years of age) female squirrel monkeys (*Saimiri boliviensis boliviensis*). Monkeys were obtained from a colony of the Squirrel Monkey Breeding and Research Resource (SMBRR) at the Michale E. Keeling Center for Comparative Medicine and Research (KCCMR), University of Texas (UT) MD Anderson Cancer Center (MDACC). The primates were socially housed in a colony and relocated in small groups of up to three per cage, based on planned treatment assignment. The animals included in the study had never been subjected to any experimental treatment and were disease free. Fourteen animals were enrolled in the study.

#### 2.1.2. Ethics Statement

This research was conducted at the AAALAC-I-accredited Michale E. Keeling Center for Comparative Medicine and Research, UT MD Anderson Cancer Center, Bastrop, TX, USA. The monkeys were examined by veterinarians before and during the study period and determined to be healthy. Experiments were approved by the Institutional Animal Care and Use Committee of the University of Texas MD Anderson Cancer Center and were carried out according to the principles included in the *Guide for the Care and Use of Laboratory Animals* [31].

### 2.2. Blood Collection

At each time point, blood (3 mL) samples were collected in EDTA anticoagulant (1.5 mL) tubes and coagulation (1.5 mL) CBC tubes, from the femoral vein. All blood sample collections occurred in the morning (8-10 AM) before the animals were fed, by utilizing manual restraints, with no sedatives as described previously [4, 32].

All animals were removed from their respective social groups and relocated into one unoccupied room, within the SMBRR colony and away from other squirrel monkeys. The blood samples were collected prior to relocation (day 0), the day of relocation (day 1), and two different days (day 6 and 15) after relocation. Monkeys were socially housed throughout the study period in two connecting cages that are 4′ wide × 6′ tall × 14′ long.

#### 2.2.1. Clinical Laboratory Measures

Routine blood hematology and biochemistry screens were performed at all time points, prerelocation (Pre) and days 1, 6, and 15 after relocation. Whole blood samples collected in EDTA tubes (BD Vacutainer) were used to measure hematology parameters using ADVIA 120 Hematology Analyzer (Siemens). Serum was separated from blood collected in tubes with no coagulant (BD Vacutainer) and used for serum chemistry on a Beckman Coulter AU680® Chemistry Analyzer as described in Nehete et al. [4].

### 2.3. Collection of Plasma Samples and Peripheral Blood Mononuclear Cells (PBMCs)

Before the separation of peripheral blood mononuclear cells (PBMCs) from whole blood samples, blood was centrifuged at 485 g × 5 min (RT), and plasma was collected and stored immediately at -80°C until analyzed. PBMCs were prepared from blood samples by standard Ficoll-Hypaque density-gradient centrifugation for various immune assays as described in Nehete et al. [33].

### 2.4. Flow Cytometry

Cell-surface markers were determined using the following fluorescence-labeled monoclonal antibodies specific to different lymphocyte subsets: T cells (CD3-FITC clone SP34-2, CD4-PerCP clone L200, and CD8-PE clone 3B5), B cells (CD20-APC clone L27), and NK cells (CD16 BV650 clone 3G8, along with CD3 antibody). All antibodies were purchased from BD Pharmingen, San Jose, CA, except CD8 antibody (Invitrogen). Monocytes and monocyte subsets were identified using CD14-AF700 clone M5E2 BioLegend, San Diego CA) and CD16 Clone 3G8 (BD). The methods of phenotype analysis of lymphocytes in peripheral blood were performed by surface staining of whole blood samples as described previously [30, 32].

### 2.5. In Vitro Mitogen Stimulation

PBMCs, freshly prepared from whole blood, were more than 90% viable, as determined by using the Nexcelom Fluorescent Cellometer automated cell counter (Nexcelom Bioscience LLC, Lawrence, MA, USA) method, and for each immune assay, 10^5^ cells/well were used. The proliferation of PBMCs was determined by the standard MTT dye reduction assay, and results are expressed as stimulation index (SI) as previously described [32, 33].

### 2.6. ELISpot Assay for Detecting Antigen-Specific IFN *γ*-Producing Cells

Freshly isolated PBMCs as described above were stimulated with the mitogens PHA, Con A, LPS, and PWM (each at 1 *μ*g/mL final concentration) to determine the numbers of IFN *γ*-producing cells by the enzyme-linked immunospot (ELISpot) assay using the methodology reported previously [4, 30, 32].

### 2.7. Cytokine Multiplex Assays

Cytokines/chemokines were measured in plasma from EDTA-preserved whole blood using Luminex technology, NHP Multiplex Magnetic Bead Panel (Millipore-Sigma; Burlington, MA), to measure IFN *γ*, IL1RA, IL1*β*, IL6, IL12 (P40), IL4, TNF*α*, and VEGF levels as described previously and according to the manufacturer's protocols [4, 30, 32]. The final levels are expressed as pg/mL, following correction for dilution factor.

### 2.8. Cortisol Measurement by ELISA

Plasma samples were collected as described earlier and frozen at -80°C until analysis. On the day of analysis, samples were thawed and clarified by centrifugation. Cortisol concentrations were measured directly, with no extraction, in diluted (1 : 50) plasma samples using an EIA kit designed for cortisol quantification in saliva (Salimetrics, Philadelphia, PA, USA). Plasma samples (25 *μ*L) diluted in an assay buffer (provided in the kit) were run as duplicates and according to the manufacturer's protocol. Resulting values were directly plotted for analysis.

### 2.9. Statistical Analysis

Details of statistical testing are provided in the result sections below. The CBC, chemistry, and immunological data are analyzed using a series of within-subject Multivariate Analyses of Variance. The primary comparisons are time postrelocation (before, immediately after, and two weeks after). In those incidences where measurements were taken repeatedly from the same subject, a repeated-measure ANOVA test was used. All statistical analyses were completed using the SPSS v26 (IBM Corp., Released 2019, Armonk, NY) and GraphPad Prism v8.4.0 (GraphPad Software, San Diego, CA USA, http://www.graphpad.com) software. The results were considered statistically significant if the tests resulted in a probability value of less than 0.05 and the Sidak corrections for testing multiple comparisons.

## 3. Results

### 3.1. Relocation of Squirrel Monkeys

**Figure 1:**
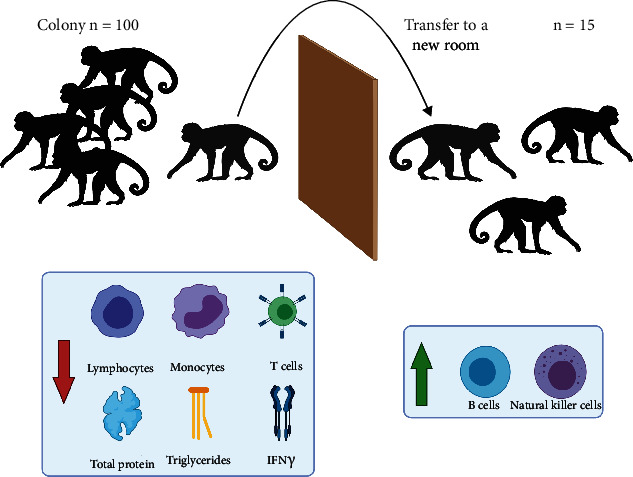
Schematic presentation of relocation of monkeys within the colony.

Schematic presentation of the relocation of monkeys within the colony is presented in Figure 1.

### 3.2. Blood Chemistry

Routine blood hematology and biochemistry screens were performed at prerelocation day and days 1, 6, and 15 postrelocation using an ADVIA 120 Hematology Analyzer (Siemens). We monitored white blood cells (WBCs), lymphocytes, monocytes, and neutrophils directly from peripheral blood. Fresh serum samples were used for serum chemistry analysis on a Beckman Coulter AU680® Chemistry Analyzer. Serum chemistry panels included measures of triglycerides, total bilirubin, alkaline phosphate, CK, total protein, globulin, cholesterol, AST, ALT, BUN, creatinine, glucose, sodium, Fe, UIBC, GGT, LDH, albumin, potassium, chloride, calcium, phosphorus, CO_2_, AGap, and osmolarity.

**Figure 2:**
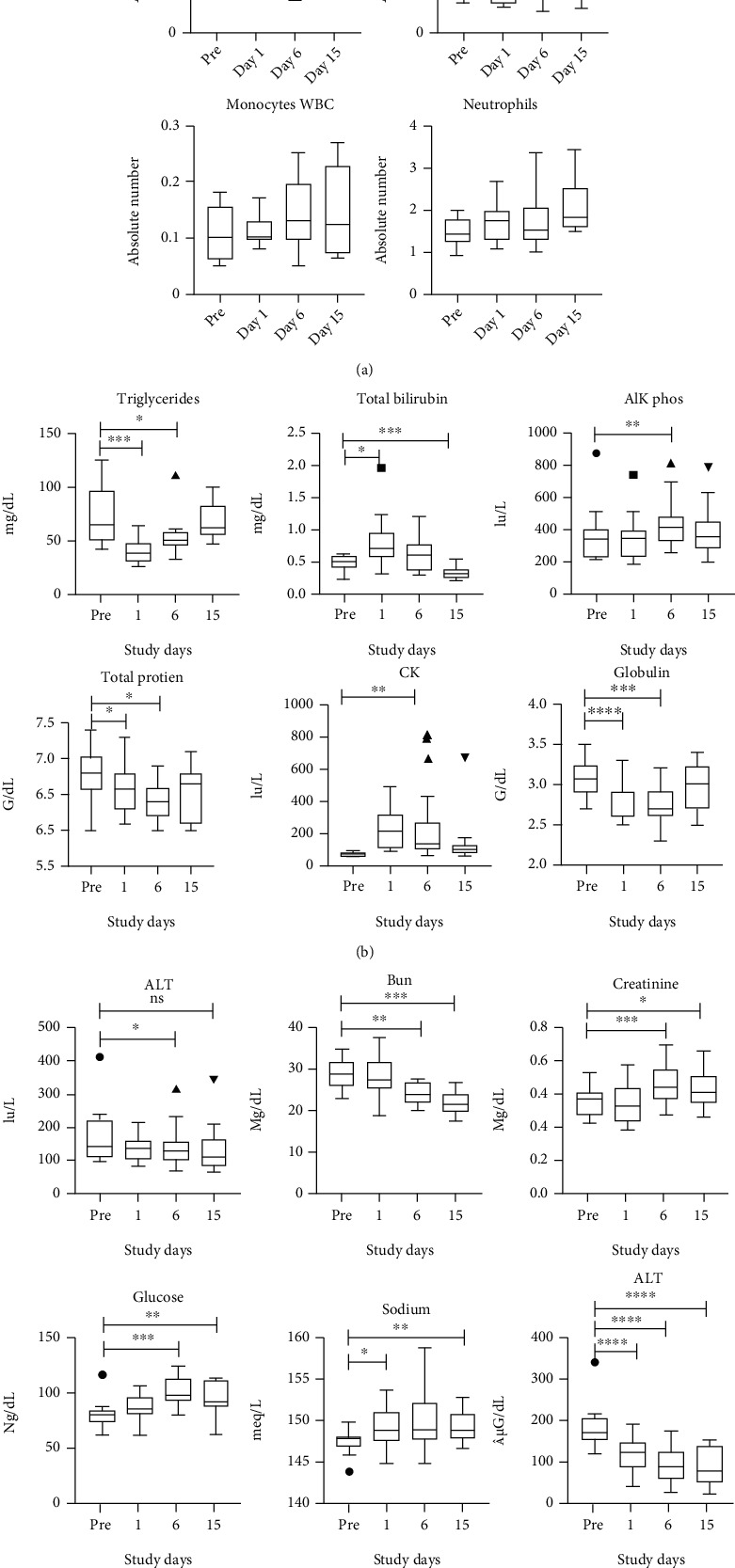
(a–c) Hematology and chemistry analysis. (a) Whole blood analyzed by using an automated analyzer ADVIA (Siemens Healthcare Diagnostics, Tarrytown, NY) at prerelocation and days 1, 6, and 15 postrelocation. Values on the *y*-axis are the absolute numbers of white blood cells (WBC), lymphocytes, monocytes, and neutrophils presented as 10^3^ per *μ*L of whole blood. (b, c) The fresh serum samples were used for serum chemistry analysis on a Beckman Coulter AU680® Chemistry Analyzer. (b) Measurement of serum chemistry panels included the measure of triglycerides, total bilirubin, alkaline phosphate, total protein, CK, and globulin. (c) Measurement of ALT, BUN, creatinine, glucose.

A multivariate ANOVA of the hematology data showed a significant effect of time (*F*(60, 90) = 42.60, *p* < 0.001, Wilk′s *λ* = 0.005, partial *η*^2^ = 0.827). Blood hematology analysis revealed significant reduction in lymphocyte numbers at days 1 and 6 postrelocation (*F*(3, 33) = 2.87, *p* = 0.004, partial *η*^2^ = 0.325) but no significant changes in WBC (*F*(3, 33) = 2.38, *p* = 0.130), monocytes (*F*(3, 33) = 2.08, *p* = 0.121), and neutrophils (*F*(3, 33) = 2.35, *p* = 0.125) (Figure 2(a)).

A multivariate ANOVA of the chemistry data showed a significant effect (*F*(3, 27) = 37.94, *p* < 0.001, partial *η*^2^ = 0.808) (*F*(75, 42) = 7.44, Wilk′s *λ* = 0.000, *p* < 0.001, partial *η*^2^ = 0.938) as shown in Figures 2(a)–2(c). Blood serum chemistry analyses revealed significant changes with pattern for triglycerides (*F*(3, 36) = 9.22, *p* < 0.001, partial *η*^2^ = 0.434), total bilirubin (*F*(3, 36) = 16.27, *p* < 0.001, partial *η*^2^ = 0.576), ALP (*F*(3, 36) = 13.10, *p* < 0.002, partial *η*^2^ = 0.522), total protein (*F*(3, 36) = 8.47, *p* < 0.001, partial *η*^2^ = 0.414), CK (*F*(3, 36) = 8.87, *p* < 0.0.01, partial *η*^2^ = 0.317), and globulin (*F*(3, 33) = 21.76, *p* < 0.001, partial *η*^2^ = 0.645) as shown (Figure 2(b)).

Blood serum chemistry revealed significant decrease at day 6 and no significant at day 15 for ALT (*F*(3, 36) = 4.39, *p* = 0.01, partial *η*^2^ = 0.268); BUN (*F*(3, 36) = 3.42, *p* = 0.028, partial *η*^2^ = 0.222), creatine (*F*(3, 36) = 22.15, *p* < 0.001, partial *η*^2^ = 0.649), glucose (*F*(3, 36) = 9.90, *p* ≤ 0.001, partial *η*^2^ = 0.452), and sodium increase at days 1 and 15 and nothing significant at day 6 (*F*(3, 36) = 4.10, *p* < 0.05, partial *η*^2^ = 0.24) and Fe (*F*(3, 36) = 35.41, *p* < 0.001, partial *η*^2^ = 0.747) (Figure 2(c)).

Relocation of study animals showed no significant pattern for cholesterol, GGT, AST, LDH, albumin, potassium, chlorine, calcium, phosphorous, CO_2_, TBIC, AGap, and osmolarity.

### 3.3. Influence of Relocation on Major Lymphocyte Subsets in the Peripheral Blood

**Figure 3:**
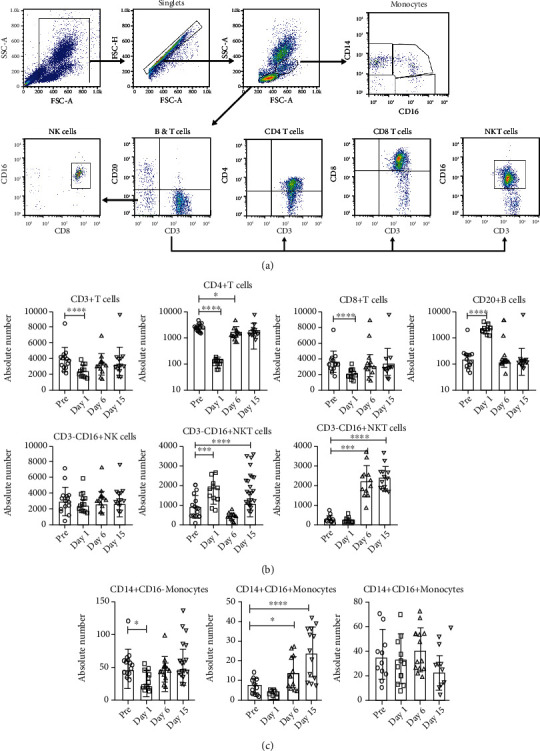
(a) Representative gating strategy showing phenotype analyses of the various cell markers in the peripheral blood. The lymphocytes and monocytes were first gated based on forward scatter (FCS) versus side scatter (SSC); CD3^+^, CD4^+^, CD8^+^, and CD20^+^ cells and NK and NKT cells were positively identified from the lymphocyte subset. Monocytes and subsets were identified from monocyte-gated population by CD14 and CD16 expression. The specificity of staining for the various markers was ascertained according to the isotype control antibody staining used for each pair of combination markers, as shown. (b) Phenotype and absolute number analysis of lymphocyte subsets from squirrel monkeys. Aliquots of EDTA whole blood were stained with fluorescence-labeled antibodies to the CD3^+^, CD4^+^, CD8^+^, CD16+, and CD20^+^ to identify lymphocyte subpopulations pre- and posttransportation and relocation. Values on the *y*-axis are absolute lymphocyte cells. *p* values were considered statistically significant at *p* < 0.05. (c) Phenotype and absolute number analysis of monocyte subsets from squirrel monkeys. Aliquots of EDTA whole blood were stained with fluorescence-labeled antibodies to the CD3^+^, CD14^+^, and CD16^+^ to identify monocyte subpopulations pre- and posttransportation and relocation. Values on the *y*-axis are absolute lymphocyte cells. *p* values were considered statistically significant at *p* < 0.05.

Phenotypic analysis of T cell subsets and B cells from the whole blood was analyzed by flow cytometry using cross-reactive human antibodies for the same antigens. The gating strategy is shown in Figure 3(a). A multivariate ANOVA revealed a significant effect of time (*F*(30, 60) = 20.61, Wilk′s *λ* = 0.000, *p* < 0.001, partial *η*^2^ = 0.927). We observed a significant decline in the absolute numbers of CD3^+^ T cells (*F*(3, 27) = 12.38, *p* < 0.001, partial *η*^2^ = 0.579), CD4^+^ T cells (*F*(3, 27) = 8.53, *p* = 0.013, partial *η*^2^ = 0.487), and CD8^+^ T cells (*F*(3, 27) = 14.91, *p* < 0.001, partial *η*^2^ = 0.624), on day 1 (morning of day immediately after relocation) postrelocation compared to prerelocation levels. This decline was transient, as absolute numbers of the same immune cells on days 6 and 15 did not differ significantly from prerelocation values, indicating a recovery from the decline seen immediately after relocation. Analysis of CD20^+^ B cells (*F*(3, 27) = 80.60, *p* < 0.001, partial *η*^2^ = 0.900), CD3^−^CD8^+^CD16^+^ NK cells (*F*(3, 27) = 83.63, *p* < 0.001, partial *η*^2^ = 0.903), and CD3^+^CD16^+^NKT cells (*F*(3, 27) = 23.01, *p* < 0.001, partial *η*^2^ = 0.719) were significantly higher 1 day after relocation and different patterns of significance on days 6 and 15 (Figure 3(b)). These analyses revealed that relocation-associated changes in the lymphocyte compartment are not uniform and inversely affect T and B cells.

We also observed significant differences in monocyte population. The classic monocyte population (CD14^+^CD16^−^) was significantly lower at day 1 postrelocation (*F*(3, 27) = 7.56, *p* = 0.011, partial *η*^2^ = 0.457). The intermediate monocyte population (CD14^+^CD16^+^) (*F*(3, 27) = 37.94, *p* < 0.001, partial *η*^2^ = 0.808) was significantly higher at days 6 and 15, while no significant changes were observed (*F*(3, 27) = 2.66, *p* = 0.109) in the nonclassic monocyte CD14^−^CD16^+^ population (Figure 3(c)).

### 3.4. Influence of Relocation on Mitogen-Induced Proliferation and IFN *γ* ELISpot Responses

As reported in human studies, relocation significantly changes immune responses, specifically in terms of functional activity of T cells in the lymphocyte compartment [34–37]. To understand the parallels in squirrel monkeys, we performed detailed analyses of cell-mediated immune responses, including (1) proliferation, (2) IFN *γ* production in response to stimulation with mitogens (e.g., Con A, PHA, PWM, and LPS), and (3) circulating levels of cytokines before and after relocation.

**Figure 4:**
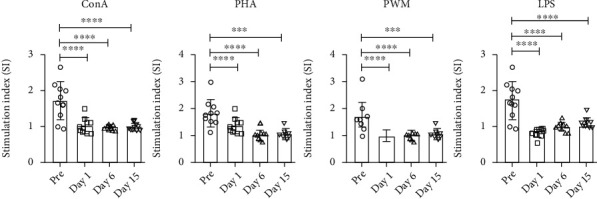
Proliferative response of PBMCs to mitogens. PBMCs that are isolated from blood samples of the squirrel monkeys were used for determining proliferative response to different mitogens, using the MTT assay. The proliferation responses are expressed as stimulation index (SI) after blank (i.e., medium only) subtraction. *p* values were considered statistically significant at *p* < 0.05.

SQM PBMCs stimulated with mitogens showed decreased proliferation after relocation. The decrease observed between prerelocation and days 1, 6, and 15 postrelocation was seen with all four mitogens (Figure 4).

A multivariate analysis of proliferative responses between pre- and postrelocation samples revealed significant changes in responses immediately following transport and relocation (*F*(12, 63) = 3.48, Wilk′s *λ* = 0.264, *p* = 0.001, partial *η*^2^ = 0.359). Significant univariate effects were seen following stimulation with Con A (*F*(3, 27) = 3.57, *p* = 0.027, partial *η*^2^ = 0.284), PHA (*F*(3, 27) = 6.82, *p* = 0.001, partial *η*^2^ = 0.431), PWM (*F*(3, 27) = 8.98, *p* ≤ 0.001, partial *η*^2^ = 0.499), and LPS (*F*(3, 27) = 6.01, *p* = 0.003, partial *η*^2^ = 0.400) mitogens. A significant decrease was observed between prerelocation and days 1, 6, and 15 postrelocation, when PBMCs were separately stimulated with the four mitogens (Figure 4).

**Figure 5:**
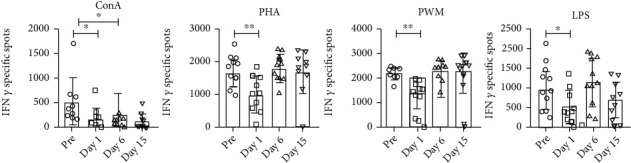
ELISpot for detecting mitogen-specific IFN *γ*-producing cells in squirrel monkeys. Triplicate wells of the 96-well microtiter plates, precoated with IFN *γ* antibody, were seeded with 10^5^ PBMCs, stimulated with 1 *μ*g of each of the mitogens for 36 h at 37°C, and then washed and stained with biotinylated second IFN *γ* antibody. The total number of spots forming cells (SFCs) in each of the mitogen-stimulated wells was counted and adjusted to control medium as background. See Materials and Methods for experiment details. *p* values were considered statistically significant at *p* < 0.05.

A multivariate analysis of PBMCs analyzed for IFN *γ* in response to stimulation with Con A, PHA, PWM, and LPS by the cytokine ELISpot assay found a significant time effect (*F*(12, 63) = 7.10, Wilk′s *λ* = 0.106, *p* < 0.001, partial *η*^2^ = 0.527). Significantly decreased responses were seen in Con A (*F*(3, 27) = 19.92, *p* < 0.001, partial *η*^2^ = 0.653), PHA (*F*(3, 27) = 16.43, *p* < 0.001, partial *η*^2^ = 0.646), PWM (*F*(3.27) = 14.01, *p* < 0.001, partial *η*^2^ = 0.609), and LPS (*F*(3, 27) = 27.45, *p* < 0.001, partial *η*^2^ = 0.753) stimulated cultures at day 1, but no differences were observed at days 6 and 15, except the significantly reduced Con A response at day 15 following relocation as compared to prerelocation responses (Figure 5).

### 3.5. Influence of Relocation on Circulating Cytokines in Plasma

**Figure 6:**
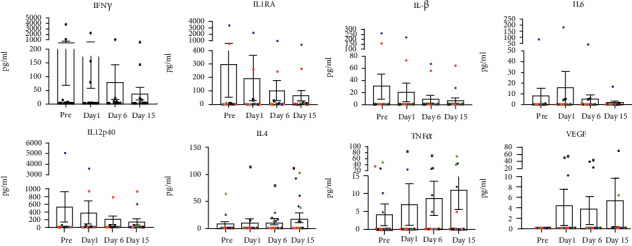
Cytokine multiplex assays. In duplicate wells of the 96-well filter plate, 25 *μ*L of cell supernatant was incubated with 25 *μ*L of cytokine-coupled beads overnight at 4°C, followed by washing and staining with biotinylated detection antibody. The plates were read on a Luminex 200 analyzer, and the results are expressed as pg/mL concentration. The minimum detectable concentrations in pg/mL for IFN *γ* (2.2), IL1RA (2.4), IL1*β* (1.2), IL6 (0.3), IL12 (P40) (1.2), IL4 (2.7), TNF*α* (2.1), and VEGF (13.6) were used for considering positive responses. See Materials and Methods for experimental details. *p* values were considered statistically significant at *p* < 0.05.

Plasma levels of cytokines/chemokines were determined using a multiplex cytokine detection kit based on the Luminex technology. Comprehensive analysis of 11 different cytokines in the plasma samples collected from squirrel monkeys (*n* = 14) did not reveal significant differences between pre- and postrelocation days in the levels of IFN *γ*, IL1RA, IL1*β*, IL6, IL12 (P40), IL4, TNF*α*, and VEGF (Figure 6).

### 3.6. Relationship between Cortisol and Immune Measurement

**Figure 7:**
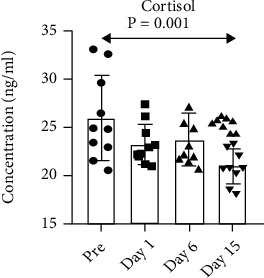
Cortisol measurement by ELISA. Cortisol concentrations in plasma were measured using an EIA kit (Salimetrics, Philadelphia, PA, USA) as described by the manufacturer. Resulting values were directly plotted for analysis.

All blood samples for cortisol analysis were collected between 8:00 and 10:00 AM. Plasma cortisol levels were lower at all time points postrelocation, but only significantly lower at day 15 (Figure 7). A one-way ANOVA with repeated-measure test revealed a significant effect of time (*F*(2, 27) = 7.03, *p* = 0.001, partial *η*^2^ = 0.44). Post hoc analysis indicated that day 15 cortisol levels were significantly lower than samples taken before the manipulation (*p* < 0.001).

## 4. Discussion

This study focused on the effects of relocation and the acclimatization period of NHP animals (squirrel monkey) within the facility as assessed by a variety of parameters that are also likely to be dependent measures in biomedical investigations. In the past, we reported similar effects in chimpanzees (1), cynomolgus macaques (2), rhesus macaques (3), and squirrel monkeys (4) relocated from out of site to our location in Texas by road transport. We suggested based on those study results to consider a period of 6-8 weeks for NHPs to adjust to their new conditions after transport, prior to their use in studies that assess the current measured parameters (immune) as dependent variables. Similar studies involving relocation of NHPs suggested acclimatization for up to three months in order to maximize the quality of research [38].

NHPs maintained in captive housing can experience a wide array of manipulations that affect their welfare and suitability as experimental subjects. Many behavioral assessments of welfare have been devised [39]. While most transport studies to date have looked at relocation between facilities, the present study investigates relocation within a facility, from one social situation to another. Capitanio et al. [40] have showed that relocation/transport of NHPs from one setting to another affects the animal's behavioral and physiological responses. These results and others have led to the recommendation that NHPs should be acclimatized for up to three months prior to experimental use to minimize interanimal variation in behavior and physiological measurements [41].

Our study establishes the physiological and immune system effects of social group relocation and acclimation for squirrel monkeys. Social relocation involves separation from familiar social partners and environments, such as changes in noises, smells, and vibrations. These negative effects have been assessed in several species [42–45], as changes in serum levels of cortisol and other physiological measures. In the present study, some standard clinical parameters of serum chemistry and hematologic values appeared to return to prerelocation levels by about day 6 after relocation, while others did not. Not all of the cell-mediated immune responses that were affected by relocation returned to prerelocation levels. The squirrel monkeys were still affected by the relocation process days after relocation and probably should not be considered acclimated to their new social environment. The effect of social stress includes impaired cellular immunity demonstrated by increased lymphocyte counts and decreased lymphocyte proliferation in response to phytohemagglutinin, as observed in cynomolgus monkeys [46–48].

We have monitored the effect of social relocation (within the facility) including temporal and environmental factors on parameters of hematology and serum chemistry combined with immune parameters and immune cell function to understand the ability of these animals to physiologically acclimate/adapt to their new surroundings. Such data will help to determine when newly arrived animals should become available for use in research studies. These responses were measured in blood samples obtained from squirrel monkeys, at different time points relative to their relocation from their old “home” to their new home previously unoccupied. These samples allowed us to measure the stress associated with transport and the amount of time required for acclimating to their new environment. A variety of basic immune assays, including analyses of lymphocyte subsets (CD4^+^, CD8^+^, CD16^+^, CD20^+^, and CD4^+^/CD8^+^), proliferation responses to mitogens (e.g., Con A, LPS, and PWM), natural killer cell responses, and cytokine production, were performed in the current study. We have used a similar battery of assays with considerable success in our examinations of the effects of dominance, enrichment, social housing, and positive reinforcement training on cell-mediated immune responses in SPF rhesus macaques and chimpanzees [1, 49, 50]. We observed transient decline in lymphocyte populations; a similar phenomenon was observed in patients with emergency medical conditions including trauma, cardiac conditions, abdominal pain, and other underlying maladies [51–53].

The changes in blood chemistry and hematology can mostly be explained as stress caused by catching of animals, blood draw, and disruption of social bonds. Creation of new social groups can have adverse effects during relocation, including changes in the RBC, Hgb, and HCT. Transient reductions in the number of WBCs have been observed with stress as part of intense physical exercise [54–56]. Variable movements of the crate may have stressed the animals, leading to the reduction in WBCs seen the day of relocation. However, some of these changes may be related to changes in some of the serum chemistry measurements of liver function.

Serum chemistry changes mostly agree with those seen hematologically. Changes in osmolality and sodium levels in squirrel monkeys may be indicative of mild dehydration. Changes in albumin, phosphorus, and triglycerides that are usually associated with kidney lesions may be indirectly related to changes in hydration. The changes seen in CK and LDH levels are usually associated with vigorous exercise. Like the fall in lymphocyes, this effect may be due to animals having to compensate for an unknown housing environment. The increase in glucose levels combined with the decreased iron levels may be attributable to an increase in overall stress. The changes seen in liver function tests (ALT, AST, and BUN and total bilirubin) are usually indicative of liver lesions. In squirrel monkeys, these changes suggest that even a small relocation has some transient effect on liver function through a yet unknown mechanism.

The short-term changes seen in some serum chemistry measurements in these squirrel monkeys may be an adjustment to their new housing conditions. Even within the same facilities, housing conditions may vary in cage materials and differing schedules for feeding, enrichment, and cleaning. These or other changes in environment and routine may be significant enough to alter animal physiology as compared to their original, prerelocation levels.

Cortisol is a steroid hormone and plays an important role in stress events, but little is known regarding the factors that could modify the capability of animals to cope with relocation events. Cortisol values typically vary dramatically by time of day, with the highest elevation between 6:00 AM and 10:00 AM [57–59]. Relocation of rhesus macaques to a new housing arrangement in a newly contracted facility resulted in self-biting behavior and sleep disturbance with elevated cortisol levels in saliva, serum, and hair [60]. Plasma cortisol concentrations were assessed before, during, and after acute stress (transfer and relocation event) in well-established social groups of squirrel monkeys. We observed decreased cortisol levels after relocation of animals in the current study. This observation, though interesting, differs from the reported increased cortisol concentrations after relocation [61, 62]. Further analysis of alternate stress hormones or factors other than cortisol may be needed to understand mechanisms underlying stress-induced changes in immune parameters.

As more effects of animal relocation are appreciated, refinements to management procedures can be made to mitigate the stress involved. Development of management techniques that take into account the effects of stress will result in enhanced welfare for the animals [63–67] and will advance our abilities to directly test experimental hypotheses, which may ultimately result in important reductions in the number of primate subjects required to efficiently test hypotheses.

Present findings allowed us to empirically measure the quantity of stress experienced by nonhuman primates as a function of being relocated within the facility from one room to another. Additionally, we were able to determine how quickly nonhuman primates acclimate to these changed conditions and whether social companions and/or living in the habitarium facilitates or disrupts acclimation. Data collected in this study allowed us to determine the most appropriate timing and conditions for relocation and housing primates, to be used in research.

The recovery of animals may depend upon duration and type of stress inducing factors. In the present study, squirrel monkeys were relocated within the colony to a small separate room with no preexisting monkeys to avoid additional stress factor of new monkeys. Thus, we observed a fast recovery of most of the parameters close to prerelocation. However, the standard for animals to recover relocation stress period varies from individual animal and species. In our past experience with a squirrel monkey colony of 500 when they were relocated from Alabama to Bastrop, Texas, 10 hrs. by road, it took about 150 days to recover from relocation stress [4].

The data presented should guide researchers in determining a suitable acclimation phase involving squirrel monkeys or other nonhuman primate species. There are additional factors, such as sex, age, genotype, health status, previous life experience, allometric differences, and even the time of year that relocation happens, that should be taken under consideration by investigators when using nonhuman primate animals as a biological model for human disease studies.

## Data Availability

The data used to support the findings of this study are available from the corresponding authors upon request.

## Abbreviations

AST: Aspartate aminotransferase
ALT: Alkaline transaminase
BUN: Blood urea nitrogen
CK: Creatine kinase
UIBC: Unsaturated iron binding capacity
GGT: Gamma-glutamyl transferase
LDH: Lactate dehydrogenase
Alb: Albumin
K: Potassium
Cl: Chloride
Ca: Calcium
P: Phosphrous
CO_2_: Carbon dioxide
AGap: Anion gap
Osm/L: Osmolarity.

## Additional Points

*Key Points*. (1) Changes in social or housing situations can affect hematological and immunological parameters in squirrel monkeys. (2) Animals that have recently experienced social or housing changes should be allowed to reacclimatize prior to use in scientific studies.
